# Intraductal papillary mucinous neoplasm of the biliary tract in the caudate lobe of the liver: a case report and literature review

**DOI:** 10.3389/fonc.2023.1114514

**Published:** 2023-07-03

**Authors:** Xunhao Zhu, Qingqiang Ni, Qianchang Wang, Chaoqun Ma, Faji Yang, Hengjun Gao, Huaqiang Zhu, Xu Zhou, Hong Chang, Jun Lu, Fangfeng Liu

**Affiliations:** Department of Hepatobiliary Surgery, Shandong Provincial Hospital Affiliated to Shandong First Medical University, Jinan, Shandong, China

**Keywords:** intraductal papillary mucinous neoplasm, bile duct, pathology, diagnosis, treatment, biliary tract surgery

## Abstract

An intraductal papillary mucinous neoplasm of the biliary tract (BT-IPMN) in the caudate lobe of the liver is a rare tumor originating from the bile duct. Approximately 40% of the intraductal papillary neoplasms of the biliary tract (IPNB) secrete mucus and can grow in the intrahepatic or extrahepatic bile ducts. A 65-year-old woman presented with recurrent episodes of right upper pain. She developed her first episode 8 years ago, which resolved spontaneously. The frequency of symptoms has increased in the last 2 years. She underwent laparoscopic hepatectomy and choledochal exploration and was pathologically diagnosed with a rare BT-IPMN of the caudate lobe after admission. Here, we review studies on IPNB cases and systematically describe the pathological type, diagnosis, and treatment of IPNB to provide a valuable reference for hepatobiliary surgeons in the diagnosis and treatment of this disease.

## Introduction

Intraductal papillary neoplasms of the bile tract (IPNB) have a low incidence and are rare in clinical practice with reports mainly concentrated in the Asian population. IPNB can be caused by intraductal calculus irritation or liver fluke infection (1–6). Studies have shown that IPNB mostly occurs in middle-aged to older adult people aged 55–65 years, with a slightly higher prevalence in males than in females (7–11). Patients commonly present with upper abdominal pain, fever, and jaundice, and asymptomatic patients are rare (12–14).

Here, we report a patient with IPNB who presented with apparent abdominal pain, nausea, and vomiting. She had no history of jaundice or chills. The patient’s preoperative tumor marker levels (carbohydrate antigen 19-9 and α-fetoprotein) were normal, and no significant abnormalities were observed in other related laboratory markers. The patient had a good outcome with no recurrence after radical surgery. We also summarize the clinicopathological characteristics, diagnosis, differential diagnosis, and treatment of intraductal papillary mucinous neoplasms of the biliary tract (BT-IPMN) in the caudate lobe.

## Case report

**Figure 1:**
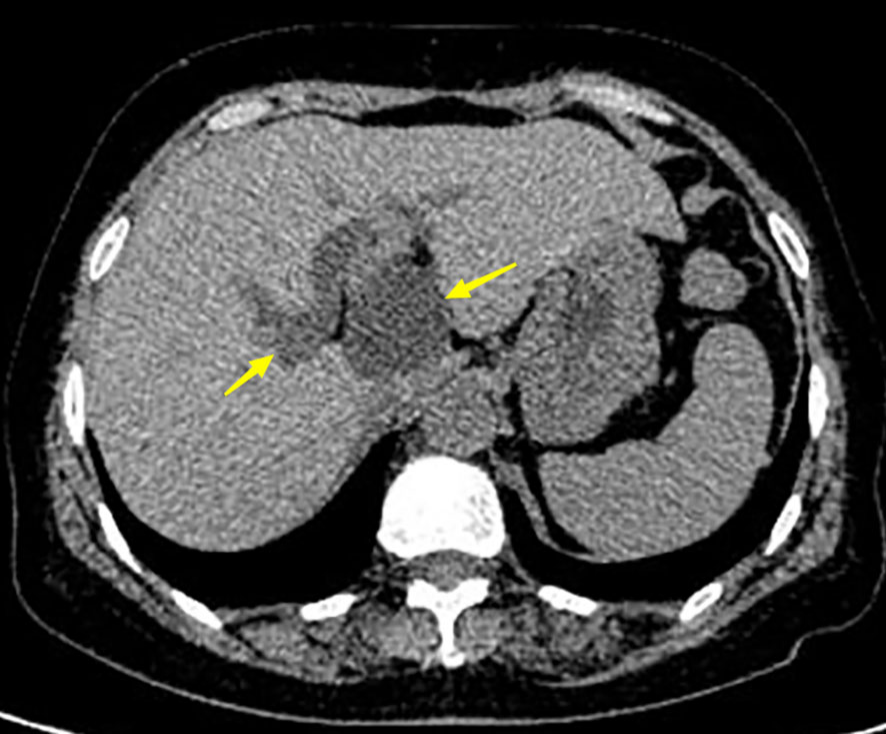
Abdominal computer tomography (CT) of the patient. Left arrow shows the dilated bile duct with a diameter of 2.1 cm. The right arrow indicates the tumor with a diameter of 4 cm.

**Figure 2:**
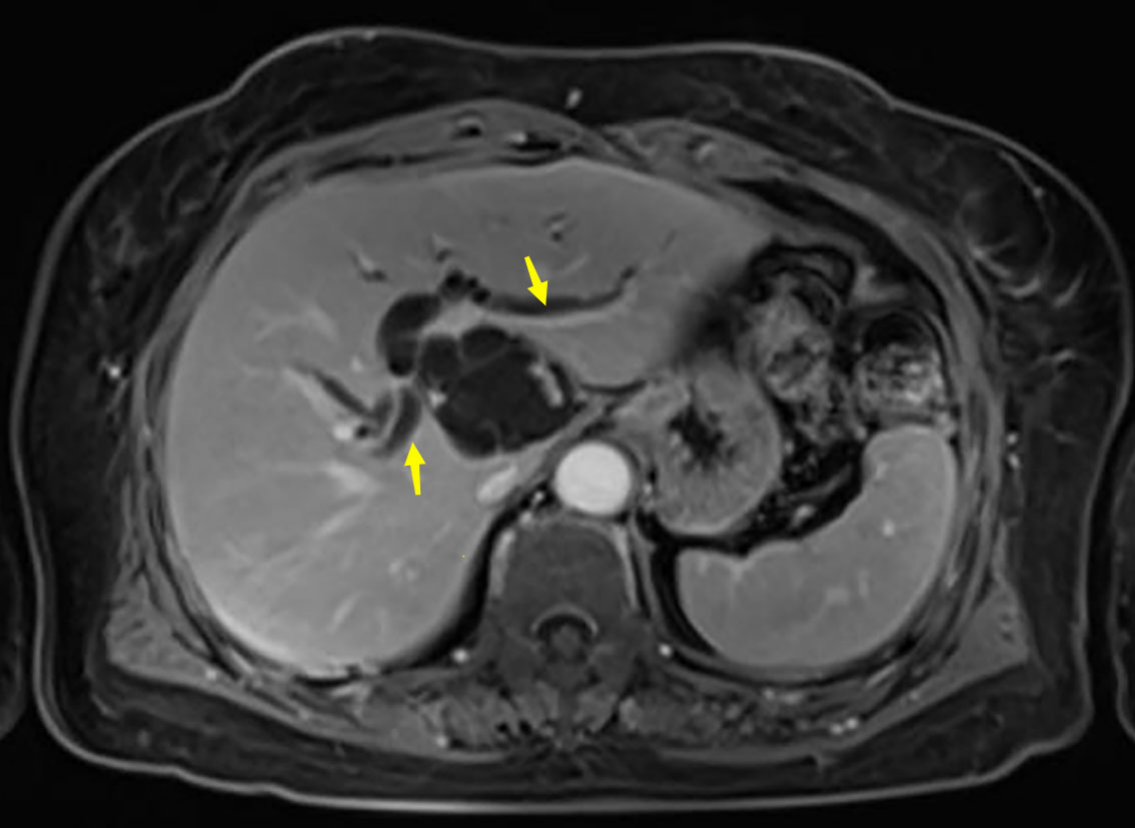
Abdominal magnetic resonance imaging (MRI) of the patient. Arrows show the dilated left and right bile ducts.

**Figure 3:**
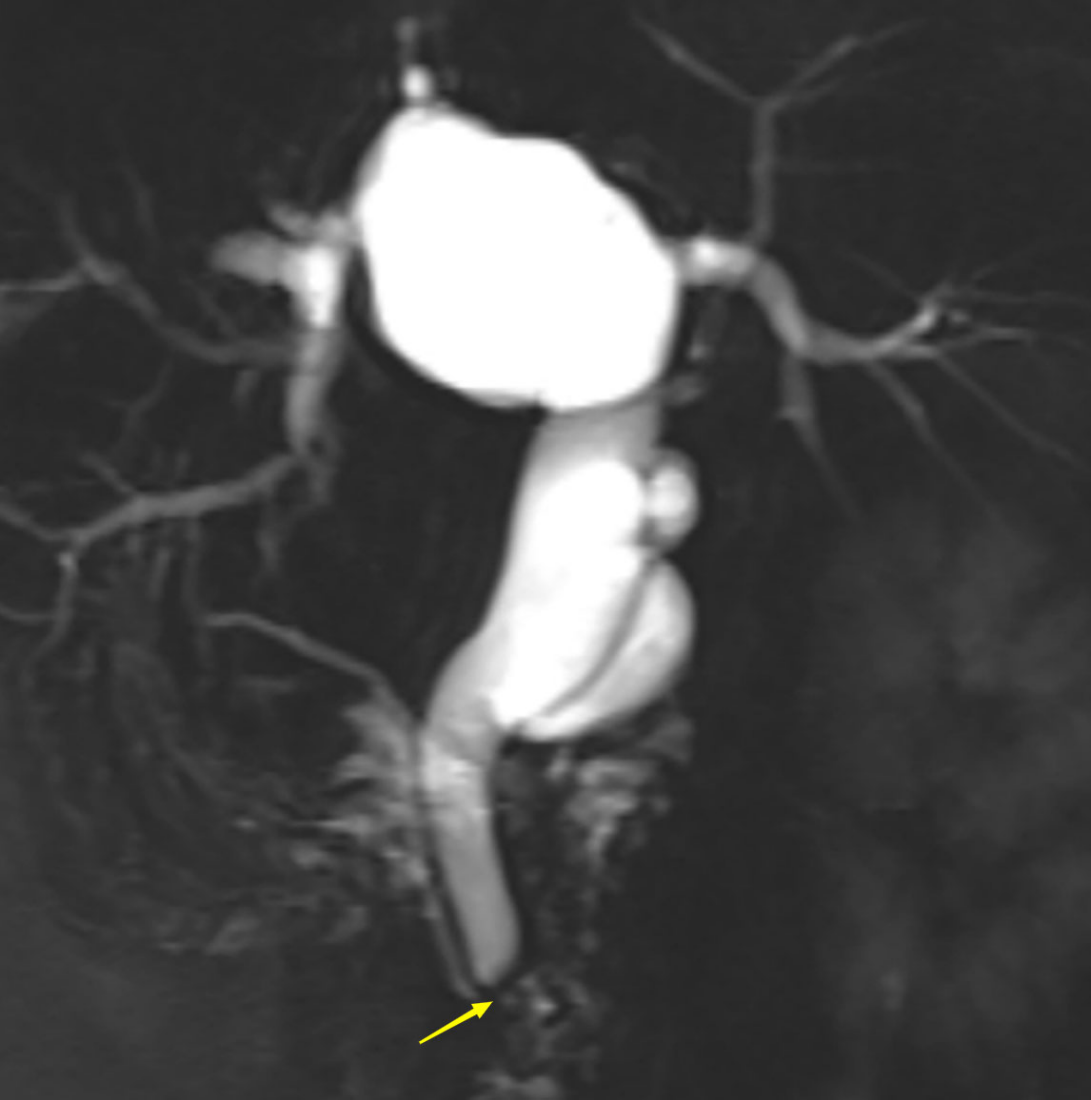
Abdominal magnetic resonance cholangiopancreatography (MRCP) of the patient. Arrow shows the blocked common bile duct.

A 63-year-old woman was admitted with a history of recurrent episodes of right upper extremity pain for more than 8 years. Eight years prior, the patient had developed abdominal pain for no apparent reason. The pain spontaneously resolved after 20 min without any treatment. She experienced abdominal pain one to two times a year, which had increased in frequency and duration over the last 2 years. Abdominal pain was accompanied by nausea and vomiting; however, fever, chills, abdominal distension, and diarrhea were absent. The patient has no history of surgery, major trauma, or any chronic diseases such as hypertension or diabetes mellitus. She denied a history of any infectious diseases, such as hepatitis and tuberculosis, food and drug allergies, smoking, or alcohol consumption. Systemic skin, mucosa, and scleral icterus were absent. Her abdomen was supple and non-tender with no rebound tenderness. Abdominal varices were absent. The liver and spleen were not palpable below the costal margins. Percussion pain in the liver region, gastric, and intestinal shapes, and peristaltic waves were not observed. Murphy’s sign was negative. Routine blood investigations, coagulation profiles, and tumor marker (carbohydrate antigen 19-9 and α-fetoprotein) levels were all normal. Computed tomography (CT) revealed intra- and extrahepatic biliary duct dilation; the maximum diameter of the common bile duct was 2.1 cm (**Figure 1**). A 4-cm diameter cystic lesion was visualized in the caudate lobe of the liver (**Figure 1**). Magnetic resonance (MR) images revealed a tumor-like tortuous dilation of the right hepatic duct at the hilum (**Figure 2**); T1WI images showed a hypointense signal, and T2WI images showed a hyperintense signal. Tubular dilation of the common bile duct and left hepatic duct in the hilum was observed (**Figure 2**). The terminal wall of the common bile duct was slightly thickened with a stenosed lumen and blockage. Enhancement of the bile duct wall was uniform and delayed. Sudden blockade of the terminal common bile duct (**Figure 3**) and nodules on the magnetic resonance cholangiopancreatography (MRCP) ductal walls were observed. The clinical diagnosis was an intrahepatic bile duct cyst.

The patient underwent laparoscopic hepatectomy and choledochal exploration on January 6, 2021, with no contraindications for surgery. The gallbladder was enlarged during surgery, the common bile duct was wider (2 cm), and a cystic mass was located in the caudate lobe of the tail of the liver protruding toward the hepatogastric space. Intraoperatively, the diagnosis was changed to hepatobiliary cystadenoma. The hepatobiliary cystadenoma was resected, and the common bile duct was explored. The common bile duct was dissected, and a 1.5-cm longitudinal incision was made along it. A jellylike liquid was aspirated from the bile duct. Choledochoscopy revealed that the lower end of the common bile duct was patent without a tumor, left hepatic duct contained a cloudy exudate, and opening of the bile duct tumor was located in the left caudate lobe. A 0.6-cm-wide bile duct converged with the left bile duct at the tumor site. The duct was resected and ligated, and tumor was completely resected and sent for histopathological examination. Rapid pathological examination revealed a mucoepithelial cyst in the caudate lobe with mild-to-moderate atypia, suggesting a mucinous cystic neoplasm.

**Figure 4:**
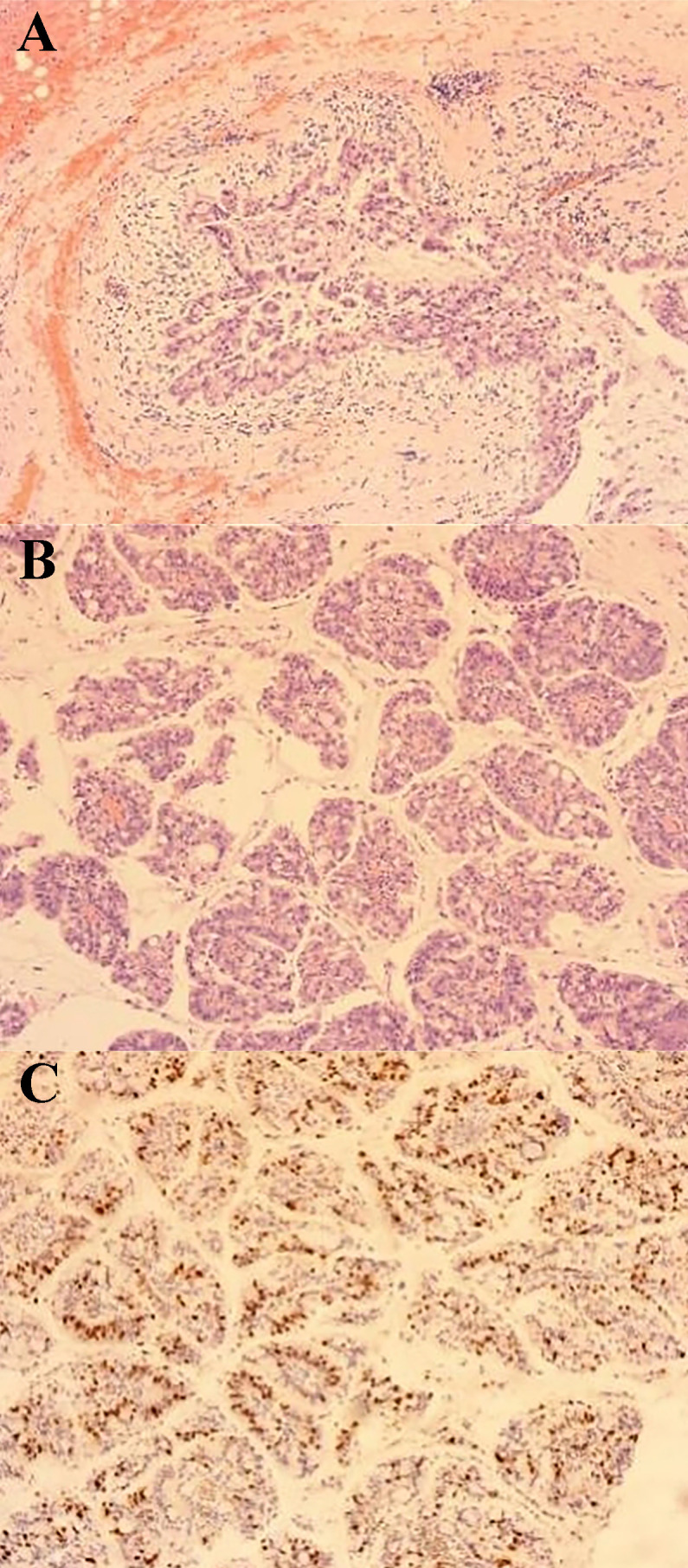
Pathological image 1 (100× magnification) **(A)** Cystic tumor: the inner wall of the cyst is lined with mucoepithelial cells showing papillary arrangement with mucus in the cyst cavity. **(B)** Papillary structure in the cyst: cytoplasmic vacuoles and papillary structure with vascular axes could be seen. **(C)** Immunohistochemistry: Ki67 proliferation index was 40% positive.

**Figure 5:**
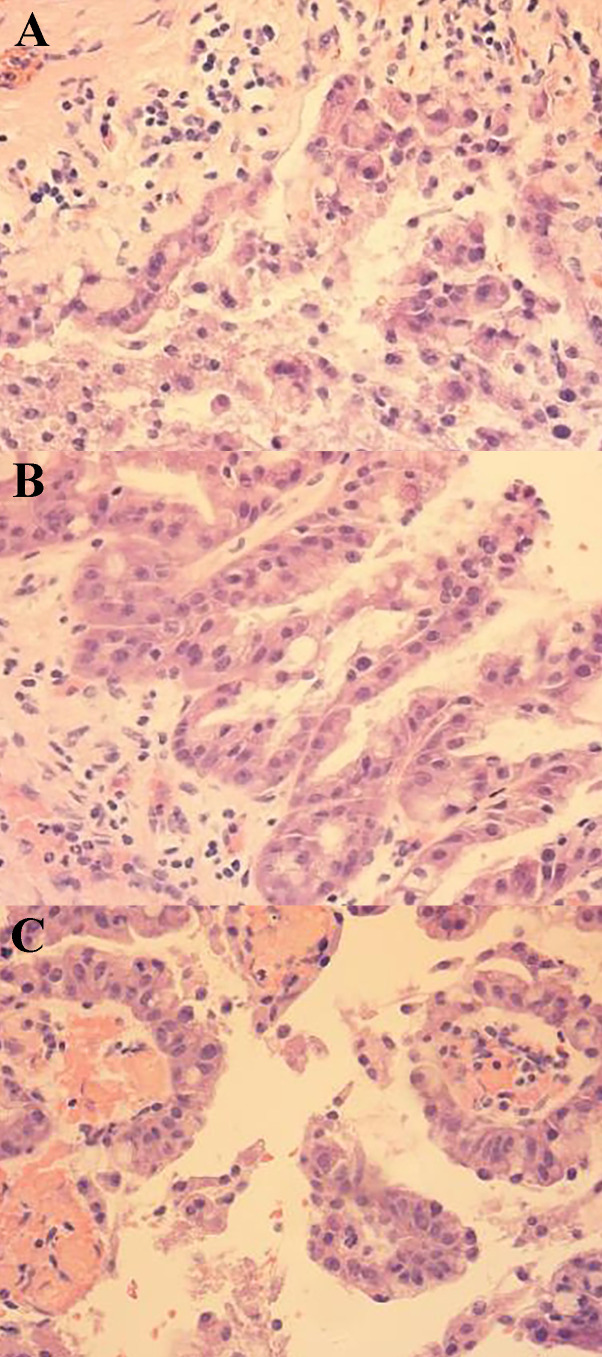
Pathological image 2 (400× magnification) **(A)** Tumor cytoplasm was stained red, which showed nuclear deviation, multisided arrangement, and mild cellular hyperplasia. **(B)** Papillary structure of the tumor. **(C)** vascular axes could be seen in the cytoplasm, which is the true papillary structure.

The surgery was successful, and the patient recovered well. She was discharged 3 days after surgery. Pathological examination of the paraffin sections (**Figures 4**, **5**) revealed an IPNB, a low-grade cystic lesion measuring 4.5 cm × 3.5 cm in volume with cells rich in mucus, which met the definition of BT-IPMN. Immunohistochemistry revealed estrogen receptor (−) and Ki-67 expression (40%). The patient visited the outpatient clinic irregularly after surgery until November 2021 to observe tumor recurrence. To date, no tumor recurrence has been observed.

## Discussion

BT-IPNB is a secretory tumor rarely observed in clinical practice. Approximately 40–80% of IPNBs are invasive cancers, either tubular or mucinous adenocarcinomas. IPNB has a high malignancy potential (6). The 5th edition of the World Health Organization (WHO) Classification of Digestive System Tumors and other related papers indicate that IPNB is a precancerous lesion of invasive cholangiocarcinoma (15–18). Related overseas studies have pointed out that a high IPNB invasion frequency is associated with the overexpression of mucin-1 and deletion of mucin-2 (19). Mucus-secreting IPNB is usually associated with carcinoma *in situ* or minimally invasive tumors, whereas non-mucus-secreting IPNB is associated with invasive tumors; greater infiltration depth is associated with poorer patient survival (20, 21).

Risk factors for IPNB include intrahepatic stones, Clonorchis sinensis infection, primary sclerosing cholangitis, bile duct malformation, familial adenomatous polyposis, and Gardner syndrome (1, 8). The global incidence of IPNB has not yet been reported. East Asian countries, such as South Korea and Japan, have the highest prevalence of IPNB with a high prevalence of intrahepatic stones and *C. sinensis* infection. The incidence of IPNB in East Asia is higher than that in Western countries (1–6).

However, the mechanism underlying the malignant transformation of IPNB remains unclear. The FGF10–RAS–ERK signaling pathway reportedly controls the ratio of dividing cells along the ductal axis resulting in rapid lung and lacrimal gland growth in developing rats (22, 23). Tomita et al. used an FGF10-induced IPNB mouse model to examine the relationship between the FGF10–FGFR2-RAS–ERK signaling pathway and IPNB occurrence and progression. Blocking the FGF10–FGFR2–RAS–ERK signaling pathway in mice inhibited FGF10-induced papillary changes and progression. FGF10-induced IPNB exhibits stepwise carcinogenesis and is associated with invasive carcinoma when mutations occur in Kras^G12D^, p53, p16, or both. The FGF10–RAS–ERK pathway is a potential novel therapeutic target for IPNB treatment (24).

**Figure 6:**
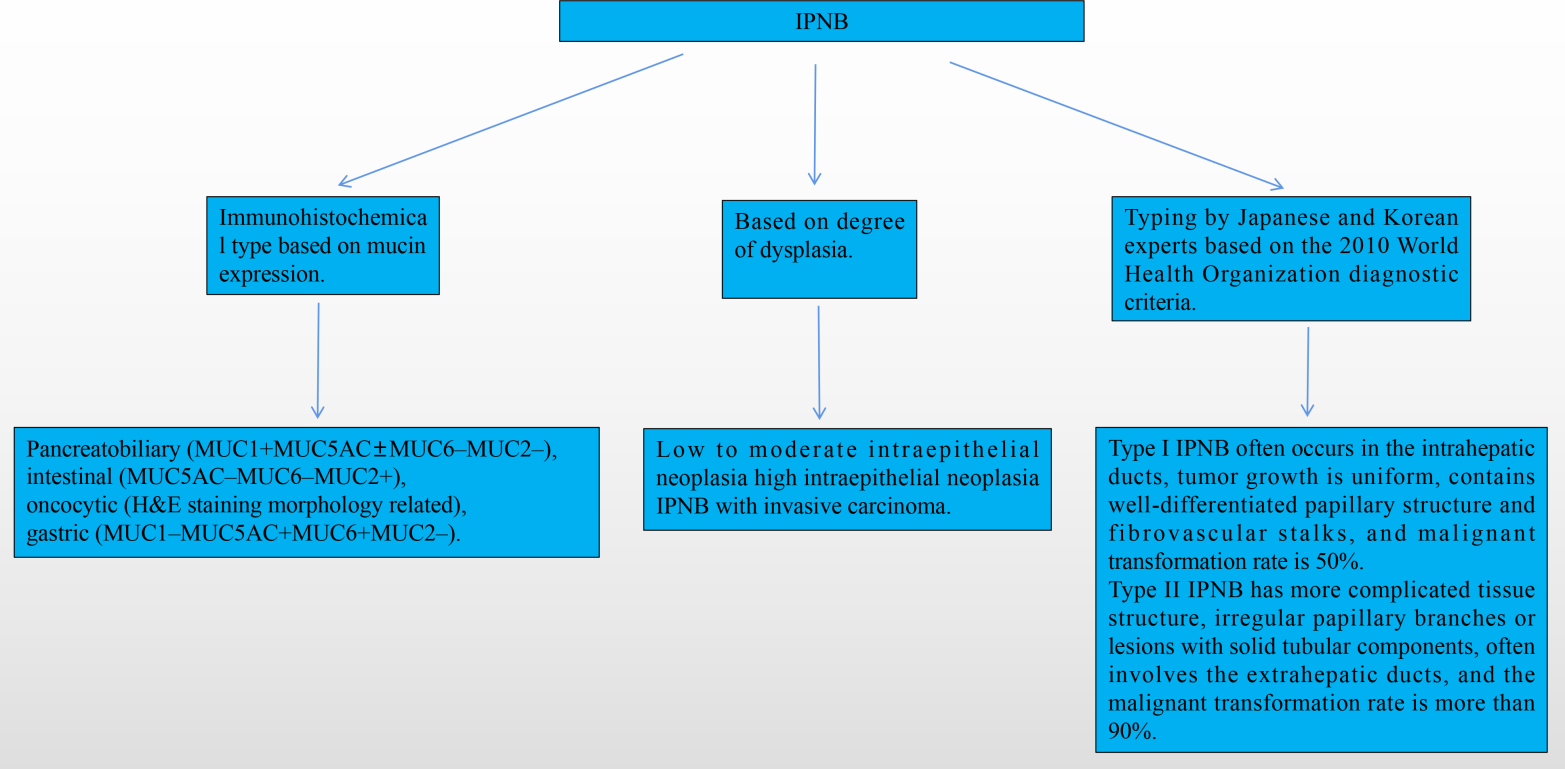
IPNB grading.

IPNB is similar to the pancreas-derived IPMN (25–27). IPNB can be divided into the following types based on the immunohistochemical analysis of mucin expression: pancreatobiliary (MUC1+MUC5AC ± MUC6−MUC2−), intestinal (MUC5AC−MUC6−MUC2+), oncocytic (H&E staining morphology-related), and gastric (MUC1−MUC5AC+MUC6+MUC2−). The intestinal type is the most common, with the most malignant behavior, whereas the gastric type has the least malignant behavior (28). IPNB can be classified as low-to-moderate intraepithelial neoplasia and high intraepithelial neoplasia; IPNB with invasive IPNB is classified based on the degree of atypical hyperplasia (24, 28). Nakanuma et al. proposed that IPNB can be divided into types I and II. Type I IPNB often occurs in the intrahepatic ducts and contains well-differentiated papillary structures and fibrovascular stalks. Type II IPNB has a more complicated tissue structure, often involving the extrahepatic ducts, with a malignant transformation rate >90% (**Figure 6**) (29). Furthermore, Kubota et al. established that the incidence of low-to-moderate and high dysplasia in patients with type I IPNB was significantly higher than that in patients with type II IPNB. However, the incidence of invasive carcinoma is higher in type II than type I IPNB. The prognoses of these two types of patients are significantly different (3). The common symptoms of IPNB include abdominal pain, jaundice, fever, and abnormal liver function. In 2015, Wang et al. reported a clinical study that included 19 patients with IPNB. More than half of the patients developed abdominal pain, 7 developed jaundice, and 16 developed cholangitis (12). Rocha et al. collected the clinical data of 39 patients who had been treated at the Memorial Sloan-Kettering Cancer Center between 1993 and 2010; 15 patients developed abdominal pain, 14 developed jaundice, 6 developed abnormal liver function tests, 2 developed cholangitis, and 2 were asymptomatic (5). Yeh et al. conducted a retrospective analysis of 122 patients with IPNB who had undergone surgery at Chang Gung Memorial Hospital between 1977 and 2003; approximately 80%, 59%, 30%, 11%, and 5% of the patients developed right-sided rib pain, fever, jaundice, anemia, and weight loss, respectively (4). Kubota et al. conducted an international multicenter clinical study that retrospectively analyzed the data of 694 pathologically confirmed cases of IPNB in patients treated in medical centers in Japan and South Korea from 1995 to 2017. Approximately 46.6%, 25%, 11.7%, and 18.6% of the patients developed liver impairment, jaundice, fever, and abdominal pain, respectively. The incidence of these symptoms was higher in patients with type II IPNB than in those with type I IPNB (3) (**Table 1**).

**Table 1:** IPNB data of the study population.

Reference	Study population	Number of patients with IPNB	Preoperative clinical factors of patients (n/N %)
Xing Wang et al., 2015 (12)	19 patients with IPNB from January 2000 to December 2013	19	Abdominal pain (15/19 78.9), Jaundice (7/19 36.8). Cholangitis (16/19 84.2)
Flavio G. Rocha et al., 2012 (5)	39 patients admitted to the Memorial Sloan-Kettering Cancer Center between 1993 and 2010	39	Abdominal pain (15/39 39) Jaundice (14/39 36) Elevated liver enzymes (6/39 15) Cholangitis (2/39 5) Asymptomatic (2/39 5)
Ta-Sen Yeh et al., 2005 (4)	122 patients underwent surgery in the Department of Surgery, Chang Gung Memorial Hospital between 1977 and 2003	122	Right hypochondriac pain (98/122 80) Fever (72/122 59) Jaundice (36/122 30)Anemia (11/122 9)Weight loss (2/39 5)
Keiichi Kubota et al., 2020 (3)	Patients treated surgically KOR (n = 406), JPN (n = 288)	694	Liver dysfunction (278/597 46.6) Fever (77/659 11.7)Jaundice (171/683 25) Abdominal pain (126/678 18.6)

As there were differences in the intraoperative and preoperative diagnoses in the patients, rapid pathological examination of the resected tumors showed mucinous cystic neoplasms (MCN). Mucinous cystic neoplasms of the liver (MCN-Ls) are generally known as biliary cystadenomas (carcinoma). However, its relationship with BT-IPMN remains unclear. At present, most people consider MCN-L and BT-IPMN as two different types of tumors (30–32). MCN is a preinvasive intraepithelial neoplasia associated with ovarian-like stroma. MCN differs from IPNB in that it does not invade the bile ducts, while MCN-L tends to occur in women, is multilocular, and has a low probability of malignant transformation. MCN and BP-IPMN have different histological characteristics and prognoses. Therefore, differentiating between these two types of tumors is important in clinical practice.

Preoperative diagnosis of IPNB is primarily dependent on radiological methods. In recent years, improvements in professional skills and continuous advancements in equipment have increased the radiological detection rates of IPNB. Currently, the most commonly used radiological methods are CT and MRI. Although ultrasonography is simple and easy to perform, its diagnosis is difficult owing to gaseous interference. Therefore, ultrasonography was not used as the main diagnostic tool. The lesions are small and difficult to discover in the early stages of the disease. The tumor secretes large amounts of mucus, bile duct dilates, ductal walls thicken, or tumor blocks the lumen as the disease progresses resulting in a characteristic presentation (1). Lisotti et al. found that contrast-enhanced harmonic endoscopic ultrasound provides increased value for the identification and characterization of malignant mural nodules (33). Facciorusso et al. established that endoscopic ultrasound-guided through-the-needle biopsy should be selectively used to evaluate patients with IPMNs (34). CT alone has limited value in the diagnosis of IPNB, as it can be confused with other diseases, such as intrahepatic stones and comorbid inflammation, which can cause biliary dilation. A recent retrospective study found that MRI has higher sensitivity and accuracy for IPNB diagnosis than CT (35). MRCP, intraoperative choledochoscopy, and intraoperative pathological examination can be used to aid diagnosis when necessary.

Patients with a confirmed diagnosis of IPNB should undergo surgery for tumor resection as soon as possible to prevent disease progression. Tumor size and site, surgical method, and resection area can be determined using preoperative radiological examinations. Choledochoscopy and pathological examination of the resected margin should be performed during surgery to identify tumor status and determine whether further resection is required. Partial hepatectomy is usually performed if the tumor invades the intrahepatic duct. Partial bile duct resection and duodenectomy can be considered in patients with extrahepatic duct invasion (3, 21). Kubota et al. established that extrahepatic IPNB is more invasive than intrahepatic IPNB and associated with a lower survival rate. Patients with negative resection margins have a better prognosis than those with positive resection margins (3). Wang et al. retrospectively analyzed the prognosis of 19 patients with IPNB; 14 patients had benign tumors, and the median overall survival was 68 months (12). Kubota et al. found that the 1-, 3-, 5-, and 10-year cumulative survival rates of patients with type I IPNB were 96.1%, 85.2%, 75.2%, and 58.5%, respectively, while the 1-, 3-, 5-, and 10-year cumulative survival rates of type II IPNB were 94.6%, 69.1%, 50.9%, and 26.8%, respectively. Patients with type I IPNB have significantly better survival rates than those with type II IPNB (3).

In conclusion, IPNB has a low incidence rate and is difficult to diagnose. Common diagnostic methods include CT and MRI. Patients with a definite diagnosis of IPNB and no contraindications to surgery should undergo surgery to attain the greatest benefits.

## Data availability statement

The original contributions presented in the study are included in the article/supplementary material. Further inquiries can be directed to the corresponding author.

## Ethics statement

The studies involving human participants were reviewed and approved by the Ethics Committee of Shandong Provincial Hospital. The patients/participants provided their written informed consent to participate in this study. Written informed consent was obtained from the individual(s) for the publication of any potentially identifiable images or data included in this article.

## Author contributions

XHZ: Writing-original draft, data curation and writing review & editing. QN: investigation, methodology, writing review & editing, conceptualization and data curation. CM: writing review & editing. HG: investigation, methodology, and writing review & editing. FY: methodology and writing review & editing. HZ: investigation, methodology, and writing review & editing. QW: investigation and supervision. XZ: investigation, supervision, and methodology. JL: conceptualization, methodology, and supervision. HC: conceptualization, methodology, and supervision. FL: investigation, methodology, conceptualization, and supervision. All authors had full access to all the data in the study and had final responsibility for the decision to submit for publication. All authors contributed to the article and approved the submitted version.
